# The role of alexithymia on psychological resilience in women with breast cancer

**DOI:** 10.1192/j.eurpsy.2021.1981

**Published:** 2021-08-13

**Authors:** F. Atkan, F. Oflaz, Z. Bahar

**Affiliations:** 1 Nursing (psychiatric Nursing), Koç University Graduate School of Health Sciences, Istanbul, Turkey; 2 Nursing (psychiatric Nursing), Koç University School of Nursing, Istanbul, Turkey; 3 Faculty Of Health Sciences, Nursing, Istanbul Kent University, ISTANBUL, Turkey

**Keywords:** psychological resilience, perceived social support, alexithymia, women with breast cancer

## Abstract

**Introduction:**

Studies indicated that breast cancer cause alexithymia that having adverse effect on resilience. Recognizing and expressing emotions are very crucial to cope with the difficulties.

**Objectives:**

This study aimed to examine the role of alexithymia on psychological resilience and related variables in women with breast cancer.

**Methods:**

In this descriptive study, 70 women with breast cancer who apply to a medical oncology outpatient between June 2019-February 2020 were included. 9-questions questionnaire was used to determine the sociodemographic and cancer related characteristics of the participants. The Multidimensional Scale of Perceived Social Support (MSPSS), Toronto Alexithymia Scale (TAS-20), Psychological Resilience Scale (PRS) were used to determine perceived social support, alexithymia and psychological resilience levels. Descriptive statistics, correlations, ANOVA and t-test were used for data analysis.

**Results:**

The MSPSS (20.07 ± 10.54) and TAS-20 were found low (47.71 ± 11.96) and PRS were high (132.24 ± 16.47). A negative, weak, significant relationship was found between the alexithymia (r=-0.370, p=0.02) and perceived social support (r=-0.496, p=0.01) with psychological resilience. There was no significant difference between the psychological resilience and age, education level, marital status, having children, profession, employment status, duration of illness, type of treatment, having metastases, and becoming caregiver (p> 0.05).

**Conclusions:**

The psychological resilience of women with breast cancer was negatively related to their alexithymia and perceived social support levels. It indicates that being able to recognize the emotions and having social support systems would positively affect the recovery process.

**Disclosure:**

No significant relationships.

